# Critical review of clinical practice guidelines for evaluation of neck mass in adults

**DOI:** 10.1016/j.bjorl.2021.03.005

**Published:** 2021-04-10

**Authors:** Kevin Chorath, Aman Prasad, Neil Luu, Beatrice Go, Alvaro Moreira, Karthik Rajasekaran

**Affiliations:** a University of Pennsylvania, Department of Otorhinolaryngology, Philadelphia, United States; b University of Texas Health-San Antonio, Department of Pediatrics, San Antonio, United States; c University of Pennsylvania, Leonard Davis Institute of Health Economics, Philadelphia, United States

**Keywords:** Neck mass, Cervical lymphadenopathy, Guideline, Consensus, AGREE II

## Abstract

**Objective:**

Several clinical practice guidelines have been produced and disseminated for the evaluation of a neck mass. However, to date, the quality and methodologic rigor of these clinical practice guidelines have not been appraised. Therefore, this study set out to identify and assess the methodologic quality of national and international guidelines for the evaluation and management of neck masses in adults.

**Methods:**

We conducted a comprehensive search of EMBASE, MEDLINE/PubMed, SCOPUS and grey literature sources until September 2020. The quality of these guidelines was assessed by four reviewers using the Appraisal of Guidelines for Research and Evaluation, 2nd edition (AGREE II). Domain scores were considered acceptable quality if they scored >60%, and Intraclass Correlation Coefficients (ICC) were calculated to assess agreement among the appraisers.

**Results:**

Seven guidelines were assessed for evaluation. Among these, only the American Academy of Otolaryngology (AAO), Cancer Care Manitoba (CCMB), and the American Society of Clinical Oncology (ASCO) achieved an overall rating of “high”. The remaining four guidelines achieved ratings of either “average” or “low”. The “Scope and Purpose” domain achieved the highest mean score (94.4% ± 5.0%), and lowest was “Applicability” (51.5% ± 29.2%). ICC analysis showed substantial to very good agreement across all domains (0.75–0.98).

**Conclusion:**

These findings highlight the variability in methodologic quality of guidelines for the evaluation and management of adult neck mass. The results from this analysis highlight the need to improve guidelines development process for this topic and may guide the selection and use of these guidelines in clinical practice.

## Introduction

It is not uncommon for patients to present to their physician with a neck mass, but identification of an underlying etiology may pose a challenge.^1^ Generally, when the mass is accompanied by signs of inflammation, short-term observation with a course of antibiotics is a reasonable option.^2^, ^3^, ^4^ However, if the mass persists or grows in size, further investigation is warranted to rule out other infectious, inflammatory, or neoplastic causes.^5^ Accordingly, a neck mass may be the only manifestation of malignancy of the head and neck, and the top priority should be ruling out this insidious cause.^2^, ^6^ Historically, many patients experienced delays in diagnosis up to 5–6 months from initial presentation of symptoms.^7^ Even with advances in imaging technology and increases in healthcare access, the delays can still range between 3–6 months.^8^, ^9^, ^10^, ^11^ This is particularly worrisome since delays in diagnosis of even 2 months have been shown to have worse outcomes and increase the chance for recurrence.^11^, ^12^

There are several important considerations when evaluating patients for a neck mass. Questions like “should I order a CT scan?”, “does this patient need a biopsy?”, or “when do I refer to a specialist?” are legitimate concerns, especially for those encountering these patients in the primary care setting. In the past 5 years, several professional societies have released Clinical Practice Guidelines (CPGs) for the evaluation and management of a neck mass, but these guidelines vary in quality, rigor of development, and/or fail to provide an easily understandable algorithm that providers can follow. The heterogeneity seen with CPGs can lead to difficulties with adaption and implementation. The Appraisal of Guidelines for Research and Evaluation II (AGREE II) is a validated tool used to assess the process of development and methodologic rigor for clinical guidelines.^13^ In fact, this tool has been used extensively to assess other clinical topics in otolaryngology including, but not limited to, head and neck imaging, pediatric hearing loss and chronic sinusitis.^14^, ^15^, ^16^ To date, there have been no studies using validated evaluation instruments to assess the methodologic rigor of CPGs relating to evaluation of neck mass. As such, the goal of this systematic review is to assess the methodologic quality of national and international guidelines for the evaluation and management of neck mass in adults.

## Methods

### Identification of guidelines

A systematic search was performed with MEDLINE (via PubMed), SCOPUS, EMBASE, and grey literature from time of inception to September 1, 2020. Additionally, we performed a manual Google search to identify guidelines not found in these databases. We used the following search terms: (“neck mass” OR “neck masses” OR “neck lesion” OR “neck lump” OR “neck swelling” OR “cervical lymphadenopathy” OR “cervical adenopathy” OR “neck growth”) AND (“recommendation” OR “guideline” OR “consensus” OR “assessment”). Two reviewers independently evaluated the search results on the basis of inclusion and exclusion criteria detailed below. The reviewers also extracted general characteristics of the guidelines. Disputes were resolved by consensus with the other members of the group.

### Selection of guideline

Every clinical guideline included in this study contains explicit recommendations on the diagnosis and evaluation of adult patients presenting with a neck mass. If societies had published multiple guidelines, we used the most up-to-date version for analysis with the AGREE II tool. Reviewers thoroughly evaluated the accompanying appendices and supporting documents for every guideline to better inform inclusion in the final list of guidelines. We excluded primary studies, systematic reviews, textbook chapters, editorials and letters, clinical trials, guidelines published in non-English language, and documents that were not available in the full-text format.

### Quality appraisal

Four authors independently assessed the clinical guidelines using the AGREE II tool. Authors were trained in the methodology and evaluation criteria of the AGREE II tool via the free, online training modules available on the AGREE website (www.agreetrust.org). The tool consists of 23 items that assess six domains: (1) Scope and purpose, (2) Stakeholder involvement, (3) Rigor of development, (4) Clarity of presentation, (5) Applicability, and (6) Editorial independence. Each domain represents a unique dimension of guideline and is scored numerically between 1 to 7. A score of 1 (strongly disagree) was assigned if the guideline provided no relevant information for this domain, and a score of 7 (strongly agree) was assigned if the quality of reporting for that domain was exceptional. The scores were calculated as percentages of maximum possible scores for each domain according to the following formula: (obtained score – minimum possible score)/(maximum possible score – minimal possible score). The standardized scores ranged from 0% to 100%. A threshold of 60% was considered satisfactory for each domain of the AGREE II tool, and a CPG was rated as “high” with 5 or more domains scored >60%, “average” with 3 or 4 domains of >60%, and “low” if ≤2 domains were >60%. Additionally, an overall score was calculated for each CPG and was reported as a mean score.

### Statistical analysis

Agreement between the four reviewers was calculated using Intraclass Coefficients (ICC) analysis with 95% Confidence Intervals. The degree of agreement among reviewers was assessed as such: minor (0.01–0.20); fair (0.21–0.40); moderate (0.41–0.60); substantial (0.61–0.80) and very good (0.81–1.00).^17^ All statistical analyses were conducted using RStudio (Boston, MA).

## Results

**Figure 1:**
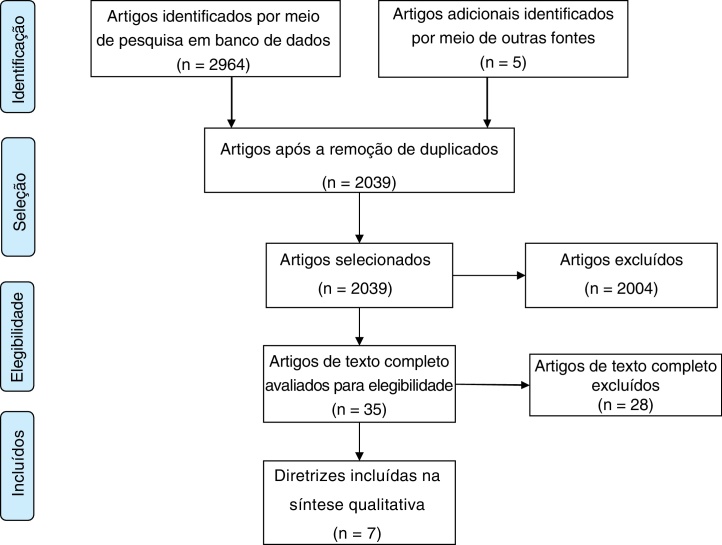
Flow diagram for identification of clinical practice guidelines and consensus statements.

The electronic search yielded 2969 titles, and 2039 records removing duplicates. After title and abstract screening, 35 documents were selected for final full text review. Ultimately, 7 CPGs met the eligibility criteria (Fig. 1).

### Guideline characteristics

CPGs were published between 2015 and 2020. Table 1 provides specific details about the development strategies, target users, number of references and any relevant funding reported in the article. Four CPGs were from United States, while the others were from Canada, France, and Australia. The development methods consisted of literature review, group consensus, and expert opinion. The development committees included otolaryngologists, family physicians, medical oncologists, radiation oncologists, pathologists, radiologists, consumer advocacy specialists and others. Funding was only explicitly reported in three guidelines.

**Table 1:** Specific details about the development strategies, target users, number of references and any relevant funding reported in the article.

Developer	Year of publication	Country/region	Development method	Developers	Target user	Number of references	Funding
American Academy of Family Physicians (AAFP)	2015	United States	Literature review	Family physicians	Family physicians and general practitioners	21	–
American Academy of Otolaryngology (AAO)	2017	United States	Literature review, expert opinion, consensus	Otolaryngologist, nurses, clinical pathologists, emergency medicine, general practitioners, consumer advocacy specialists, general surgeons, head and neck surgeons, oral and maxillofacial surgeons, physician assistants and radiologists	Otolaryngologists, family physicians, dentists, emergency physicians, pathologists, and radiologists	117	AAO
American College of Radiology (ACR)	2019	United States	Literature review, expert opinion, consensus	Radiologists, radiation oncologists, otolaryngologists	Radiologists, radiation oncologists, general practitioners, otolaryngologists	106	–
American Society of Clinical Oncology (ASCO)	2020	United States	Literature review, expert opinion, consensus	Otolaryngologists, surgical oncologists, radiation oncologists, pathologists, medical oncologists, radiologists, patient advocacy specialists	Medical oncologists, radiation oncologists, surgeons, radiologists, pathologists, nurses, speech pathologists, oncology pharmacists, and patients	178	ASCO
Cancer Care Manitoba (CCMB)	2015	Cancer	Literature review, expert opinion, consensus	Otolaryngologists, nurses, epidemiologists, pathologists, radiologists, general surgeons, and medical oncologist	Otolaryngologists, radiation therapists, family physicians, nurses, radiation oncologists, pharmacists	88	CCMB
French Society of Otorhinolaryngology-Head and Neck Surgery (SFORL)	2019	France	Literature review, expert opinion,	Otolaryngologists, radiologists	Otolaryngologists, radiologists	60	–
Royal Australian College of General Practitioners (RACGP)	2020	Australia	Literature review	Family physicians	Family physicians, general practitioners, otolaryngologists	25	–

### Quality assessment of CPGs

The overall ICC obtained for each domain is presented in Table 2. The ICC calculated for the CPG assessment using the AGREE II tool suggests overall agreement among the appraisers. The mean quality scores for each domain as well as the overall quality of each guideline is presented in Table 3.

**Table 2:** The overall ICC obtained for each domain.

Guideline	Domain 1	Domain 2	Domain 3	Domain 4	Domain 5	Domain 6	Overall score (average)	Overall quality
	Scope and purpose %	Stakeholder involvement %	Rigor of development %	Clarity and presentation (%)	Applicability %	Editorial independence %		
AAFP	84.7	55.6	50.0	97.2	35.4	50.0	62.2	Low
AAO	98.6	70.8	90.6	97.2	85.4	54.2	82.8	High
ACR	94.4	55.6	84.9	95.8	47.9	100.0	79.8	Average
ASCO	100.0	97.2	94.3	100.0	90.6	95.8	96.3	High
French	90.3	36.1	44.3	97.2	22.9	50.0	56.8	Low
Australia	94.4	22.2	19.3	72.2	8.3	100.0	52.7	Average
Manitoba	98.6	90.3	93.8	84.7	69.7	100.0	89.5	High
Mean + SD	94.4 ± 5.0	61.1 ± 25.2	68.2 ± 27.8	92.0 ± 9.3	51.5 ± 29.2	78.6 ± 23.6	–	–

**Table 3:** Intraclass coefficients for AGREE II domains.

Agree II domain	Intraclass correlation coefficient	95% confidence interval
Scope and purpose	0.75	0.18–0.95
Stakeholder involvement	0.98	0.93–0.99
Rigor of development	0.98	0.96–1.00
Clarity of Presentation	0.93	0.76–0.99
Applicability	0.97	0.90–0.99
Editorial independence	0.98	0.95–1.00
Agree II domain	Intraclass correlation coefficient	95% confidence interval
Scope and purpose	0.75	0.18–0.95
Stakeholder involvement	0.98	0.93–0.99
Rigor of development	0.98	0.96–1.00
Clarity of Presentation	0.93	0.76–0.99
Applicability	0.97	0.90–0.99
Editorial independence	0.98	0.95–1.00

### Scope and purpose

This domain evaluates the objectives of the study and the patient population to whom the guideline is applied. The mean score for this domain was 94.4 ± 5.0 and all CPGs were rated above 60%.

### Stakeholder involvement

This domain addresses whether the development process included all relevant stakeholders and also expresses the views and preferences of the target population. The mean score was this domain was 61.1 ± 25.2, and only three CPGs scored above 60%.

### Rigor of development

This domain focusses on methods used to derive the specific recommendations in CPGs and whether risks and benefits of the interventions have been considered. This domain achieved a mean score of 68.2 ± 27.8, of which four guidelines scored above 60%.

### Clarity and presentation

This domain determines whether the recommendations are specific and unambiguous, are easily identifiable, and whether different options are presented for practitioners to consider. The mean score for this domain was 92.0 ± 9.3, and all CPGs achieved greater than 60%.

### Applicability

This domain is related to implementation and execution of the guideline into real clinical practice and includes important components like facilitators and barriers to practice, resource implications, and monitoring and auditing strategies. The mean score for this domain was 51.5 ± 29.2, and only two guidelines achieved satisfactory performance.

### Editorial independence

This domain appraises the views and competing interests of the funding bodies/developing members and whether they may have influenced the final recommendations reported in the guidelines. The mean score for this domain was 78.6 ± 23.6, and four guidelines scored greater than 60%.

### Overall CPG assessment

Among the CPGs evaluated, only the American Academy of Otolaryngology (AAO), Cancer Care Manitoba (CCMB), and the American Society of Clinical Oncology (ASCO) achieved an overall rating of “high”. The remaining four guidelines achieved ratings of either “average” or “low”.

## Discussion

Early detection and timely intervention are especially important for the management of head and neck cancers, and health professionals must use a combination of clinical judgement, patient factors and high-quality evidence to best direct care for these patients. Clinical practice guidelines are useful tools designed to assist practitioners and help direct patients in making important decisions and improving clinical outcomes.^18^, ^19^, ^20^ Advantages include reducing variability in clinical practice, incorporating recommendations based on multidisciplinary discussion and high-quality evidence, and supporting the most cost-effective approach.^21^, ^22^ In fact, a prior study showed that using guidelines for the management of oral cavity cancer resulted in improved outcomes for these patients.^23^ On the other hand, poorly developed guidelines may not be easily applicable in the clinical setting or may be heavily biased with little supporting evidence. The present study is the first to assess the quality and methodologic rigor of CPGs for the evaluation of an adult neck mass. We identified seven guidelines from across the world and determined that only three CPGs were of high quality, and the others would benefit from further optimization and transparency in reporting.

This analysis demonstrated that the “Scope and Purpose” and “Clarity of Presentation” domains achieved the highest overall rating. All guidelines achieved satisfactory performance for these domains and adequately addressed the objectives of the guidelines, target populations, and provided clear recommendations. Importantly, several guidelines also described different imaging options available to the practitioners, including computed tomography (CT) and ultrasound, that can provide details on the size, location, consistency, and extent of the mass.

Conversely, only three guidelines achieved satisfactory performance for the “Stakeholder involvement” domain. The diagnosis and management of an adult neck mass requires a multidisciplinary team as the etiology of a neck mass can be widely varied. Having a team that includes otolaryngologists, family medicine physicians, radiologists, pathologists, and other practitioners is helpful as each person in the team provides a different viewpoint and skillset regarding patient presentation, diagnosis, and treatment. For instance, radiologists can help delineate the radiologic appearances for benign and malignant tumors.^24^, ^25^, ^26^ Similarly, pathologists are the ones who provide the diagnosis and can therefore describe the merits of a fine needle aspiration versus excisional biopsies in the diagnosis of various disease processes.^27^, ^28^ Unfortunately, we noted that several guidelines failed to incorporate these important professionals in the committee groups for these CPGs.

In an online survey of guideline appraisers using the AGREE II tool, the “Rigor of Development” and “Editorial Independence” domains were considered to have the strongest influence on overall CPG quality.^29^ For this study, only 43% and 57% of CPGs achieved satisfactory performance for the “Rigor of Development” and “Editorial Independence” domains, respectively. These findings likely explain the scarcity of “high” quality guidelines for this topic. Several guidelines neither reported a criterion for selecting evidence, nor provided an explicit link between the recommendations and the supporting evidence. Similarly, a statement regarding the role of the funding body or competing interests by development group members was not reported by several guidelines. Omission of these important components renders concern about the validity of the recommendations and whether any external factors could directly or indirectly undermine the objectivity of the recommendations. Future guidelines may consider including details on the systematic review used to gather evidence, benefits/side effects of specific diagnostic strategies, procedures for updating the guidelines, and any relevant monetary relationships.

Perhaps the most important aspect about an adult neck mass is determining if it represents a malignancy. As stated, prior, survival and quality of life for head and neck cancer is directly related to the size at first detection.^30^ One of the challenges in diagnosing these cancers is that many of the symptoms are also associated with other common, benign conditions.^11^ As a result, the “Applicability” domain is arguably the most important for implementation of guidelines to ensure that at-risk individuals receive the appropriate workup and consideration for this potentially deadly disease. Regrettably, this domain achieved both the lowest average score as well as the greatest variability in scores. It is well recognized that race and socioeconomic factors significantly affect head and neck cancer outcomes.^31^ For example, compared to Caucasians, African American patients are more likely to be diagnosed at a later stage and have worse disease-free and overall survival rates.^32^ Since this domain is concerned with facilitators and barriers to application, we would have liked to see increased emphasis on racial, socioeconomic and educational barriers in understanding and navigating a potential cancer diagnosis. Similarly, there is other important information that can be elicited from patient history which can increase suspicion for malignancy. This includes assessing for the presence of voice changes, tonsillar asymmetry, or ipsilateral otalgia. In the AAO guidelines, a quick reference guide and patient pamphlet was provided that practitioners could reference when evaluating patients with this condition. Unfortunately, very few other CPGs provided tools or accompanying materials that individuals could use to augment their practice.

The results from this critical appraisal are consistent with the AGREE II analysis for several other diseases. In an analysis of ten international guidelines for the diagnosis and management of chronic rhinosinusitis, only half were reported to be of good or sufficient quality.^16^ Likewise, the existing guidelines for the treatment of oral cavity cancer were demonstrated to be suboptimal, with only three out of twelve guidelines rated as recommended and an overall “Applicability” domain score of 32.2%.^33^ Our study determined that the guidelines by AAO and ASCO were among the highest quality and adequately addressed each component of the AGREE II tool. The most likely explanation for why the CPGs AAO and ASCO achieve the highest quality is that they are based on step-by-step manuals on methodology and components of transparent guidelines.^34^, ^35^ These manuals cover important topics like process of development, systematic review, organizing an expert panel, and deriving recommendations, which are all essential aspects addressed in the AGREE II checklist. Future guidelines may consider referring to manuals like this to direct the development and organization of their recommendations.

There are several limitations to this study. Although we performed an exhaustive search, it is possible that we have missed some guideline not indexed on these medical databases, particularly those not in the English language. The purpose of the AGREE II tool is to assess the quality and methodologic rigor of these CPGs; however, this tool cannot assess the validity or veracity of the individual recommendations, which is beyond the scope of this study. There is a degree of subjectivity and variability when using this AGREE II tool, but some concerns for rater bias can be ameliorated by the good intraclass reliability among all reviewers. Finally, all the guidelines that were included in this analysis were from developed countries. The burden and incidence of head and neck cancer in developed countries can be different than developing nations, particularly with respect to regional differences in tobacco/alcohol consumption, betel quid chewing, and HPV/EBV exposure.^36^, ^37^, ^38^ Furthermore, patients in low-resource countries may not have access to the same studies and interventions as developed countries.^39^, ^40^ Patient compliance and followup may also be less reliable and more variable.^41^ Future efforts should be directed towards developing guidelines that can be applied in low-resource settings with consideration of these important factors.

## Conclusion

The overall quality of clinical practice guidelines for the evaluation of neck masses in adults are suboptimal, with only three guidelines rated highly and recommended for clinical use. The results from this analysis highlight the need to improve guidelines development process for this topic. Accordingly, these guidelines would benefit from inclusion of important healthcare providers, increased rigor of development, and additional consideration for implementation in the clinical setting.

## Funding

This research did not receive any specific grant from funding agencies in the public, commercial, or not-for-profit sectors.

## Conflicts of interest

The authors declare no conflicts of interest.
